# Physical activity and sleep pattern in relation to incident Parkinson’s disease: a cohort study

**DOI:** 10.1186/s12966-024-01568-9

**Published:** 2024-02-14

**Authors:** Li-Hua Chen, Shi-Yu Sun, Guijie Li, Xiang Gao, Weifeng Luo, Haili Tian, Xuanhao Zhang, Xi Yin, Ziwei Liu, Guo-Chong Chen, Guangfei Xu, Tong Liu, Fu-Rong Li

**Affiliations:** 1 Department of Nutrition and Food Hygiene, School of Public Health, Nantong University, Nantong, China; 2 Institute of Pain Medicine and Special Environmental Medicine, Nantong University, 9 Seyuan Road, Chongchuan District, 226019 Nantong, China; 3 Department of Nutritional Sciences, The Pennsylvania State University, 16801 State College, PA USA; 4 Department of Nutrition and Food Hygiene, School of Public Health, Institute of Nutrition, Fudan University, Shanghai, China; 5 Department of Neurology and Clinical Research Center of Neurological Disease, The Second Affiliated Hospital of Soochow University, Suzhou, China; 6 School of Exercise and Health, Shanghai University of Sport, Shanghai, China; 7 Department of Nutrition and Food Hygiene, School of Public Health, Suzhou Medical College of Soochow University, Suzhou, China; 8 School of Public Health and Emergency Management, Southern University of Science and Technology, 1088 Xueyuan Avenue, Fuguang community, Taoyuan Street, Nanshan District, Shenzhen, China

**Keywords:** Physical activity, Sleep pattern, Joint association, Parkinson’s disease, UK Biobank

## Abstract

**Background:**

How physical activity (PA) and different sleep traits and overall sleep pattern interact in the development of Parkinson’s disease (PD) remain unknown.

**Objective:**

To prospectively investigate the joint associations of PA and sleep pattern with risk of PD.

**Methods:**

Included were 339,666 PD-free participants from the UK Biobank. Baseline PA levels were grouped into low (< 600 MET-mins/week), medium (600 to < 3000 MET-mins/week) and high (≥ 3000 MET-mins/week) according to the instructions of the UK Biobank. Healthy sleep traits (chronotype, sleep duration, insomnia, snoring, and daytime sleepiness) were scored from 0 to 5 and were categorized into “ideal sleep pattern” (≥ 3 sleep scores) and “poor sleep pattern” (0–2 sleep scores). Hazard ratios (HRs) and 95% confidence intervals (CIs) of PD were estimated by Cox proportional hazards models.

**Results:**

During a median of 11.8 years of follow-up, 1,966 PD events were identified. The PD risk was lower in participants with high PA (HR = 0.73; 95% CI: 0.64, 0.84), compared to those with low PA; and participants with ideal sleep pattern also had a lower risk of PD (HR = 0.78; 95% CI: 0.69, 0.87), compared to those with poor sleep pattern. When jointly investigating the combined effect, participants with both high PA and ideal sleep pattern had the lowest risk of incident PD (HR = 0.55; 95% CI: 0.44, 0.69), compared to those with low PA and poor sleep pattern; notably, participants with high PA but poor sleep pattern also gained benefit on PD risk reduction (HR = 0.74; 95% CI: 0.55, 0.99).

**Conclusions:**

Both high PA and ideal sleep pattern were independently associated with lower risk of developing PD, and those with both high PA level and ideal sleep pattern had the lowest risk. Our results suggest that improving PA levels and sleep quality may be promising intervention targets for the prevention of PD.

**Supplementary Information:**

The online version contains supplementary material available at 10.1186/s12966-024-01568-9.

## Introduction

Parkinson’s disease (PD) is a progressive neurodegenerative disorder that affects 2–3% of the population over 65 years of age worldwide [1]. The main features of PD encompass motor dysfunction, including resting tremor, rigidity, bradykinesia and postural instability. Although tremendous progress has been made in the development of treatments for PD, such as pharmacological dopamine substitution (L-DOPA treatment) and deep brain stimulation, none of these treatments is curative. Considering the limited effectiveness of current available treatments, identification of modifiable lifestyle factors for PD prevention is imperative [2, 3].

Epidemiologic studies have suggested the important role of several lifestyle factors, such as physical activity (PA), in PD development [4–7]. For example, a meta-analysis summarizing 8 studies revealed an inverse dose-response association between PA and PD risk among men but not women [8]. However, the enrolled studies did not take into account sleep-related factors [9–16]. In fact, sleep issues are tightly correlated to PD [17], and deterioration of sleep quality has been demonstrated as markers of the prodromal phase of PD [18]. Indeed, previous investigations have indicated that specific sleep characteristics were linked to the incidence of PD [19–21]. Sleep-related factors include circadian rhythm, sleep duration, insomnia, and snoring, etc. Yet, few studies have simultaneously considered these various sleep traits as a whole in relation to PD risk. In addition, behavioral risk factors may not act independently and their combined effects need to be considered. To date, however, there have been few reports exploring the combined effects of PA and sleep quality on the risk of developing PD. Therefore, understanding the multifactorial combinations involving PA and different domains of sleep characteristics in relation to the development of PD will be crucial for informing effective PD prevention strategies.

In this study, we aimed to address a critical gap in the current literature by exploring the combined effects of PA and sleep quality on the risk of developing PD. By delving into the independent and joint associations of PA and overall sleep quality in the UK Biobank cohort, our study may provide valuable insights into the complex interrelationships influencing PD risk, paving the way for more effective preventive strategies in the future.

## Methods

### Study population

The UK Biobank is a large prospective cohort study established to provide a resource for investigation of the genetic, environmental, and lifestyle factors associated with a wide range of diseases [22]. The full UK Biobank study protocol is available online (https://www.ukbiobank.ac.uk/media/gnkeyh2q/study-rationale.pdf). In brief, between 2006 and 2010, approximately 500,000 ethnically diverse men and women aged 37–73 years were recruited from 22 centers across England, Wales, and Scotland. At recruitment, participants provided a wide range of information on health and diseases and underwent various physical measurements. The UK Biobank study was approved by the National Information Governance Board for Health and Social Care and the National Health Service North West Multicenter Research Ethics Committee (REC reference for UK Biobank 11/NW/0382). All participants provided informed consent through electronic signature.

### Ascertainment of PD

PD was ascertained by the algorithmic combinations of coded information from hospital admissions (diagnoses and procedures), death registries and self-reported medical condition. The detailed definition of PD was provided in Supplementary Table S1. The UK Biobank study conducted a validation study on the accuracy of code sources for identification of PD cases, which achieved a positive predictive value (PPV) of 0.91 (95% CI, 0.83, 0.96) when combining all sources (hospital admission records, death records and self-report). More information can also be found elsewhere (https://biobank.ndph.ox.ac.uk/showcase/field.cgi?id=42033). Follow-up time was calculated as the time interval between the date of baseline assessment and the censor date, diagnosis, death, or lost to follow-up, whichever came first.

### Exposures

Total PA was quantified using the modified short-form International Physical Activity Questionnaire (IPAQ), which assessed the duration and frequency of PA in walking, moderate and vigorous activity. Weekly PA was summarized using weekly total metabolic equivalent task (MET), calculated by multiplying the MET value of activity by the number of PA minutes per week. Total PA (combination of walking, moderate intensity and vigorous intensity activities) was categorized as low (< 600 MET-mins/week), medium (600 to < 3000 MET-mins/week) and high (≥ 3000 MET-mins/week) according to the instructions of UK Biobank (https://biobank.ndph.ox.ac.uk/showcase/refer.cgi?id=540) and previous studies conducted using the UK Biobank data [23, 24]. Details on classifying three levels of PA by the UK Biobank are provided in the Supplementary Table S2.

For sleep pattern, a sleep score including five sleep characteristics was applied based on previous studies [25, 26]. Healthy sleep characteristics referred to early chronotype, adequate sleep duration (7–8 h/day), not usually insomnia, no snoring, and no frequent daytime sleepiness. Supplementary Table S3 provided a detailed questionnaire and definition of each item. Participants were scored from 0 to 5, according to their number of the healthy sleep characteristics and were categorized into two groups: “ideal sleep pattern” (≥ 3 sleep scores) and “poor sleep pattern”(0–2 sleep scores).

### Covariates

As PD development may involve multiple factors, our research considered a range of covariates, including demographic and socioeconomic variables (age, sex, ethnicity, Townsend deprivation index), anthropometric measures, and lifestyle factors (BMI, smoking, sedentary time, healthy diet habits, coffee and tea intake, and alcohol consumption). Chronic conditions and medication use, such as SBP, DBP, hypertensive medication, and diabetes, were also taken into account. These variables were collected at baseline by a touchscreen questionnaire and nurse-led interviews. The Townsend deprivation index was generated based on 4 socioeconomic variables (unemployment, non-car ownership, non-home ownership, and household overcrowding) [27]. Total sedentary hours were quantified by summing up the time spent on television watching, using a computer (not at work), and driving in every 24 h. The healthy diet score was generated by using the several dietary factors as follows [28]: red meat intake less than three times each week (median); vegetable intake at least four tablespoons each day (median); fruit intake of at least three pieces each day (median); fish intake of at least four times each week (median); cereal intake of at least five bowls each week (median); and urinary sodium concentration less than 68.3 mmol/L [29]. Each favorable diet factor was assigned a score of 1 point, resulting in a total diet score ranging from 0 to 6. Coffee or tea intake was assessed at baseline using a touchscreen questionnaire [30]. Participants were asked, “How many cups of coffee do you drink each day (including decaffeinated coffee)/How many cups of tea do you drink each day (including black and green tea)?” Participants who selected “Less than one” were classified as those who never drink coffee or tea. Alcohol consumption (gram per day) was calculated based on a previous study of the UK Biobank [28]. Diabetes was defined using self-report, diagnosis codes and a glycated haemoglobin (HbA1c) level ≥ 48 mmol/mol (6.5%). Body mass index (BMI) was calculated using measured weight and height (kg/m^2^). Baseline blood pressure data were obtained by a digital blood pressure monitor (Omron HEM-7015IT; OMRON Healthcare Europe B.V., Hoofddorp, Netherlands). Mean systolic blood pressure (SBP) and diastolic blood pressure (DBP) were calculated from two automated or two manual measurements by trained nurses [29].

### Statistical analysis

For the present study, we excluded participants (1) who had PD at baseline (*n* = 697), (2) who withdrew from the study (*n* = 367), or (3) without data for our major PA and sleep exposures (*n* = 160,274). Finally, a total of 339,666 participants remained for the main analysis (Supplementary Figure S1).

Baseline participants’ characteristics were summarized across categories of PA, sleep pattern scores or PD status, and data were reported as mean ± standard deviation or percentage where appropriate. Kaplan-Meier curves were generated for PA and sleep categories in relation to risk of PD, and log-rank tests were utilized to compare different groups. Multivariate Cox proportional hazards models were used to estimate hazard ratios (HRs) and 95% confidence intervals (CIs) for PD across PA, individual sleep characteristics, and sleep pattern categories. The reference group was defined as those with low PA levels or poor sleep characteristics/pattern. A restricted cubic spline curve from a Cox regression was employed to graph the HRs for incident PD, treating PA or sleep score as continuous variables. The restricted cubic spline was implemented following Harrell’s recommendations with three knots positioned at the 10th, 50th, and 90th percentiles of the exposure variables [31]. The model was initially adjusted for age and sex, and the fully-adjusted model included age, sex, race/ethnicity (White, Asian or Asian British, Black or Black British, mixed), Townsend deprivation index, smoking status (current, previous or never), BMI, SBP, DBP, antihypertensive medication use, healthy diet score, sedentary time, alcohol consumption, coffee consumption, tea consumption and diabetes. Most of the covariates were complete, with less than 4.0% missing. For participants with missing data in the adjustment categorical variables, we put them in a separate ‘unknown’ group to preserve the original data structure and simplify the future analysis. To handle missing data in continuous variables, we used multiple imputation with chained equations [32], generating five imputations for the missing data. As PA and sleep characteristics may mutually influence each other, we ensured their mutually adjustment in the models. This approach allows for the independent assessment of the association between PA and sleep traits. We also examined the joint association of PA levels and sleep pattern with risk of PD, with the reference group being the highest-risk participants (participants had both low PA and poor sleep pattern). Additionally, we further explored the joint associations of each individual sleep characteristics and PA in relation to the risk of incident PD. Interactions between the PA levels and sleep traits on risk of PD were tested by the likelihood ratio test, by comparing the model with and without an interaction term between PA and sleep pattern/characteristics. To test the robustness of the joint associations, we conducted sensitivity analyses among individuals without any missing values for the covariates. In addition, since insomnia appeared to be not associated with incident PD, we excluded this sleep trait from the sleep pattern in the sensitivity analyses. Finally, considering the possibility of the reverse causation between early PD with decreased PA or sleep quality, we also performed sensitivity analysis by excluding participants who developed PD within the first 2 year of follow-up.

All analyses were performed with Stata (version 15.1; StataCorp). All *P* values were 2-sided and *P* < 0.05 was considered statistically significant.

## Results

### Baseline characteristics

Baseline information on exposures and covariates were collected during the initial assessment visit (2006–2010). The proportions of participants for low PA, medium PA, and high PA were 18.5%, 50.6%, and 30.9%, respectively. Additionally, 13.3% of the participants were categorized into the “poor sleep pattern”, while 86.7% fell into the “ideal sleep pattern” group. Compared to individuals with low level of PA, those with higher levels of PA were more likely to be male, White, have a lower BMI, nonsmoker, spend less time in sedentary activities, have a healthier diet, and have fewer chronic diseases, such as hypertension and diabetes. Similar trends were found in participants with an ideal sleep pattern compared to those with a poor sleep pattern. Notably, participants with higher levels of PA were with higher proportion of ideal sleep pattern; meanwhile, among individuals with an ideal sleep pattern, there were higher levels of PA in comparison to those with poor sleep pattern (Table 1). Additionally, participants who developed PD during follow-up tended to be older, male, non-smokers, hypertensive medication users, and diabetic. They also exhibited lower levels of PA and poorer sleep quality (Supplemental Table S4).

**Table 1:** Baseline participants’ characteristics by PA and sleep pattern

	PA^*^				Sleep patterns^†^	
	Low PA (18.5%)	Medium PA (50.6%)	High PA (30.9%)		Poor sleep pattern (13.3%)	Ideal sleep pattern (86.7%)
Age, y	55.8 ± 7.9	56.2 ± 8.1	56.7 ± 8.2		56.5 ± 7.8	56.2 ± 8.2
Men	29,678 (47.3%)	78,349 (45.6%)	51,529 (49.1%)		22,692 (50.3%)	136,864 (46.5%)
White	58,815 (93.8%)	163,076 (94.8%)	100,257 (95.5%)		42,358 (93.9%)	279,790 (95.0%)
Townsend deprivation index	-1.4 ± 3.1	-1.5 ± 3.0	-1.4 ± 3.0		-1.0 ± 3.2	-1.5 ± 3.0
BMI, kg/m^2^	28.5 ± 5.4	27.1 ± 4.5	26.8 ± 4.3		28.9 ± 5.3	27.1 ± 4.5
Current smoker	7,663 (12.2%)	15,715 (9.1%)	11,040 (10.5%)		6,763 (15.0%)	27,655 (9.4%)
PA/Sleep pattern						
Low/Poor	11,343 (18.1%)	21,398 (12.4%)	12,363 (11.8%)		11,343 (25.2%)	51,366 (17.4%)
Medium/Ideal	51,366 (81.9%)	150,609 (87.6%)	92,587 (88.2%)		21,398 (47.4%)	150,609 (51.1%)
High/-	-	-	-		12,363 (27.4%)	92,587 (31.4%)
Sedentary time, h/d	5.4 ± 2.7	4.7 ± 2.3	4.7 ± 2.3		5.4 ± 2.7	4.7 ± 2.4
SBP, mmHg	138.8 ± 19.3	139.1 ± 19.6	140.4 ± 19.7		140.1 ± 19.1	139.3 ± 19.7
DBP, mmHg	82.8 ± 10.8	82.1 ± 10.7	82.0 ± 10.6		83.1 ± 10.7	82.0 ± 10.7
Healthy diet score	2.6 ± 1.4	3.0 ± 1.4	3.1 ± 1.4		2.7 ± 1.4	3.0 ± 1.4
Alcohol consumption, g/d	14.6 ± 20.0	15.0 ± 18.0	16.0 ± 20.1		17.7 ± 23.6	14.8 ± 18.2
Never drink coffee	14,755 (23.5%)	35,669 (20.7%)	23,759 (22.6%)		7,360 (16.3%)	42,206 (14.3%)
Never drink tea	10,167 (16.2%)	24,117 (14.0%)	15,282 (14.6%)		10,385 (23.0%)	63,798 (21.7%)
Hypertensive medication use	14,848 (23.7%)	33,800 (19.7%)	19,465 (18.6%)		11,695 (25.9%)	56,418 (19.2%)
Diabetes	5,393 (8.6%)	9,370 (5.5%)	4,939 (4.7%)		4,184 (9.3%)	15,518 (5.3%)

Continuous variables are described as means ± standard deviation, and categorical variables are described as numbers and percentages

BMI, body mass index; DBP, diastolic blood pressure; PA, physical activity; SBP, systolic blood pressure

^*^ PA was categorized as low (< 600 MET-mins/week), medium (600 to < 3000 MET-mins/week) and high (≥ 3000 MET-mins/week)

^**†**^ Participants were scored from 0 to 5, according to their number of the healthy sleep characteristics and were categorized into two groups: “ideal sleep pattern” (≥ 3 sleep scores) and “poor sleep pattern”(0–2 sleep scores)

### PA levels, sleep pattern, and incident PD

During a median of 11.8 years of follow-up, 0.59% of the included participants (*n* = 1,996) developed PD. Supplemental Figure S2 shows the Kaplan-Meier curves for incident PD by PA and sleep pattern (all *P* for log-rank test < 0.001). The fully adjusted HRs of PD were 0.91 (95% CI: 0.81, 1.02) for those having a medium PA level and 0.73 (95% CI: 0.64, 0.84) for those having a high PA level, compared with those with a low PA level (Table 2). Compared with participants with poor sleep pattern, the fully adjusted HRs of PD were 0.78 (95% CI: 0.69, 0.87) for those having an ideal sleep pattern (Table 2). When modeling PA levels or healthy sleep scores as continuous variables, the risk of PD generally decreased monotonically across the entire range of exposures (Supplementary Figure S3). We further investigated the associations between individual sleep characteristics and incident PD. Kaplan-Meier curves regarding each individual sleep characteristic in relation to PD were shown in Supplementary Figure S4. We found that, except for “not usually insomnia”, all other factors associated with healthy sleep were significantly linked to a lower risk of PD in expected directions, with HRs ranging from 0.68 to 0.91 (Supplementary Table S5). For participants with a poor sleep pattern, the HRs for incident PD were 0.87 (95% CI: 0.67, 1.13) for those with a medium level of PA and 0.77 (95% CI: 0.57, 1.04) for those with a high level of PA, as compared with those with a low level of PA. For participants with an ideal sleep pattern, the HRs of incident PD were 0.92 (95% CI: 0.80, 1.04) and 0.73 (95% CI: 0.63, 0.84) in those with medium and high levels of PA, respectively, when comparing with participants with a low level of PA (Table 3).

**Table 2:** Hazard ratios for the associations of healthy sleep category and physical activity with risk of Parkinson’s disease

PA*	**Low**	**Medium**	**High**
Events/person-y	402/721,692	1,042/1,994,946	552/1,214,446
Age and sex-adjusted [HR (95% CI)]	Reference	0.89 (0.80, 1.00)	0.72 (0.63, 0.82)
Multivariable-adjusted [HR (95% CI)]	Reference	0.91 (0.81, 1.02)	0.73 (0.64, 0.84)
Sleep pattern*	**Poor**	**Ideal**	-
Events/person-y	336/517,961	1,660/3,413,123	
Age and sex-adjusted [HR (95% CI)]	Reference	0.77 (0.68, 0.87)	
Multivariable-adjusted [HR (95% CI)]	Reference	0.78 (0.69, 0.87)	

Multivariable model was adjusted for age, sex, ethnicity, Townsend deprivation index, smoking status, BMI, SBP, DBP, antihypertensive medication use, sedentary time, healthy diet score, alcohol consumption, coffee consumption, tea consumption, and diabetes

* PA and sleep pattern were mutually adjusted in the analyses

CI, confidence interval; HR, hazard ratio; PA, physical activity

**Table 3:** Hazard ratios for the associations of healthy sleep pattern with risk of Parkinson’s disease, stratified by physical activity

	Events/person-y	Age-adjusted [HR (95% CI)]	Multivariable-adjusted [HR (95% CI)]
**Poor sleep pattern**			
Low PA	93/128,804	Reference	Reference
Medium PA	160/246,567	0.83 (0.64, 1.07)	0.87 (0.67, 1.13)
High PA	83/142,589	0.73 (0.54, 0.98)	0.77 (0.57, 1.04)
**Ideal sleep pattern**			
Low PA	309/592,887	Reference	Reference
Medium PA	882/1,748,379	0.91 (0.80, 1.03)	0.92 (0.80, 1.04)
High PA	469/1,071,857	0.72 (0.62, 0.83)	0.73 (0.63, 0.84)

Multivariable model was adjusted for age, sex, ethnicity, Townsend deprivation index, smoking status, BMI, SBP, DBP, antihypertensive medication use, sedentary time, healthy diet score, alcohol consumption, coffee consumption, tea consumption, and diabetes

CI, confidence interval; HR, hazard ratio; PA, physical activity

### Joint association of sleep pattern and PA with incident PD

**Fig. 1:**
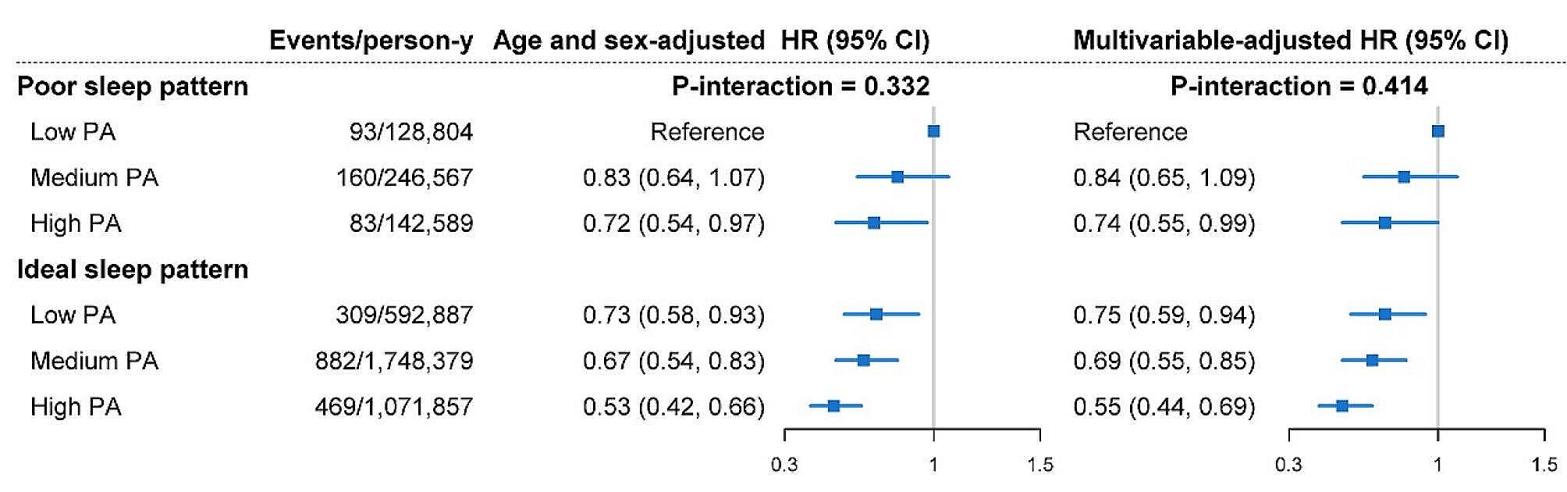
Hazard ratios for the joint associations of PA and healthy sleep category with risk of Parkinson’s disease Multivariable model was adjusted for age, sex, ethnicity, Townsend deprivation index, smoking status, BMI, SBP, DBP, antihypertensive medication use, sedentary time, healthy diet score, alcohol consumption, coffee consumption, tea consumption, and diabetes The graphs include null effect lines at HR = 1. *P*-interaction represents interaction between PA and sleep pattern for PD incidence CI, confidence interval; HR, hazard ratio; PA, physical activity; PD, Parkinson’s disease

**Fig. 2:**
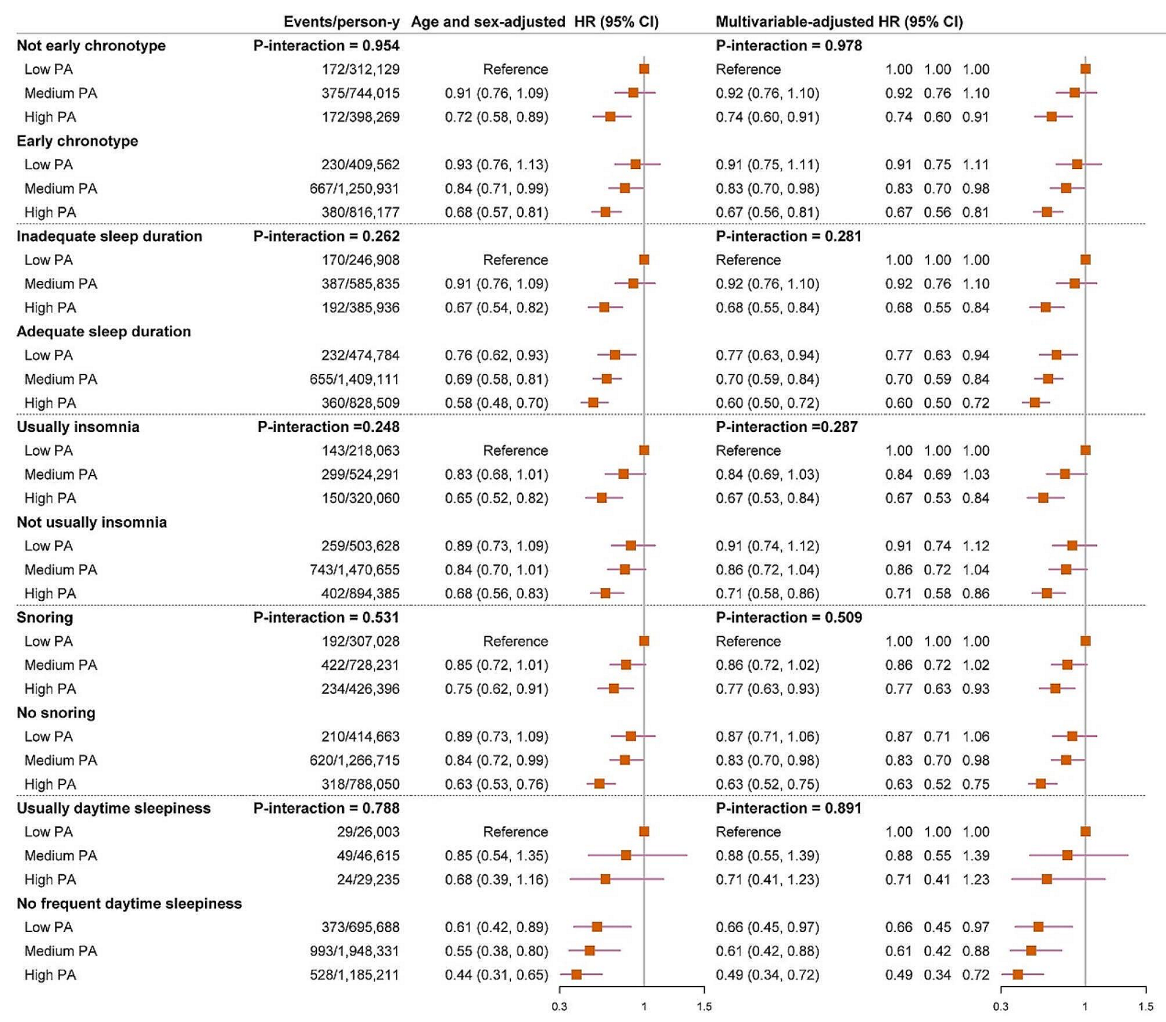
Hazard ratios for the joint associations of PA and individual sleep traits with risk of Parkinson’s disease Multivariable model was adjusted for age, sex, ethnicity, Townsend deprivation index, smoking status, BMI, SBP, DBP, antihypertensive medication use, sedentary time, healthy diet score, alcohol consumption, coffee consumption, tea consumption, and diabetes The graphs include null effect lines at HR = 1. *P-*interaction represents the interaction between PA and individual sleep traits for PD incidence CI, confidence interval; HR, hazard ratio; PA, physical activity; PD, Parkinson’s disease

Overall, we did not find any interactions between PA and sleep pattern or individual sleep characteristics (all *P*-interactions > 0.100), suggesting that PA and sleep traits may not have a mutual influence in relation to PD risk (Fig. 1). However, as compared with participants who had a low level of PA and poor sleep pattern, those with a high level of PA but poor sleep patterns may still gain benefit in lowering the risk of PD (HR, 0.74; 95% CI: 0.55, 0.99). Kaplan-Meier curve (Supplemental Figure S5) indicated that those with both high PA and ideal sleep pattern were with the lowest risk of PD (log-rank test *P* < 0.001), with adjusted HR of 0.55 (95% CI: 0.44, 0.69) (Fig. 1). We further examined whether there were sex- or age-specific joint association, but found that the joint results were largely similar across different sex or age stratum, with no evidence of interaction (*P* for interaction = 0.231 or 0.358, respectively) (Supplementary Table S6). We also examined how PA and each specific sleep characteristics were jointly associated with the risk of developing PD. Our findings showed that individuals who had a high level of PA and any type of sleep traits had a lower risk of developing PD (Fig. 2). The joint association of PA and sleep pattern were not materially changed in a serious of sensitivity analyses (Supplementary Table S7).

## Discussion

In this large population-based prospective study with 1,966 incident cases of PD among 339,666 UK participants, we found that a high level of PA was associated with a lower risk of developing PD, even among individuals with poor sleep pattern. The participants with both high PA level and healthy sleep pattern had the lowest incident PD risk. These associations were generally consistent among both sexes and across age groups.

In recent decades, a series of studies have provided profound evidence that behavioral factors may play a key role in the pathogenesis and progression of PD [33]. Among these behavioral factors, PA has been suggested as a protective factor for PD by animal studies [34–38]. The potential mechanisms for this neuroprotective effect of PA may include the increase in serum urate and the release of neurotrophic factors such as brain-derived neurotrophic factor and cerebral dopamine neurotrophic factor, and regulation of dopamine turnover [34–38]. Although a previous meta-analysis of 8 prospective studies revealed an inverse dose-response association between PA and the risk of developing PD among men, the results from some specific individual prospective studies were not statistically significant [9–16]. The discrepant results in previous studies may be attributed to variations in the measured domains of PA across these studies. For example, two studies recorded PA in recreational/leisure time only [12, 14], while the sum of household, commuting activity, occupational activity and leisure time exercise was used in another study [11], which showed that there was an inverse association with PD for total PA. Our data extended this type of investigation to a large population-based prospective cohort study with a relatively accurate information on PA and found that participants with a high total PA level (sum of work, transportation, domestic chores and gardening and leisure-time, ≥ 3000 MET-mins/week) was to be associated 27% lower incident PD risk when comparing with those with a low total PA level (< 600 MET-mins/week), after extensive adjustments for possible confounders.

Sleep disorders are common non-motor symptoms in patients with PD. However, current published studies about PA and PD did not take into account the effects of different sleep traits on risk of incident PD [9–16]. A previous prospective population-based Rotterdam Study with 5,450 participants found that poor sleep quality (evaluated by Pittsburgh Sleep Quality Index) as well as short sleep duration were linked to a higher PD risk [18]. Hsiao et al. included 91,273 participants from Taiwan National Health Insurance Research Database and found that chronic insomnia was associated with a higher risk of developing future PD [39]. Also, a cohort based on Honolulu-Asia Aging Study reported that excessive daytime sleepiness was associated with risk of subsequent development of PD [19]. Furthermore, a recent cohort study demonstrated that reduced circadian rhythmicity was associated with an increased risk of incident PD [20]. These studies, however, focused on either one or two characteristics of sleep only. Compared with other population-based studies, the current study extended this work with comprehensive analyses of 5 sleep straits/characteristics (chronotype, sleep duration, insomnia, snoring, daytime sleepiness). When considering multicomponent of sleep characteristics, a sleep score capturing aforementioned 5 sleep domains was applied; and we found that individuals with an ideal sleep pattern had a 22% reduction in the risk of incident PD after mutually adjustment for PA and other confounding factors.

It’s noteworthy that a singular lifestyle factor related to PD development is unlikely to operate in isolation; hence considering the combined effects of multiple lifestyle factors is essential. In the present study, when jointly investigating the combined effect, we found that participant with a high level of PA provide a protective effect against the development of PD, even among individuals with poor sleep patterns. Furthermore, participants with high level of PA and healthy sleep pattern had the lowest risk when compared with those in the low PA-poor sleep pattern group; and these associations were generally the same among men and women or among younger adults and older adults. Although the statistical analysis did not reveal a significant multiplicative interaction between PA and sleep, the findings may still suggest a potential additive effect of these two lifestyle factors on reducing PD risk.

Our study has some strengths. To the best of our knowledge, this is the first study to explore the association of the joint effects of PA and overall sleep quality on PD risk among the UK population. Additionally, the relatively large sample size and long-term follow-up with inpatient diagnosis of PD enabled us to perform sub-analyses with a high statistical power. However, several limitations of this study should also be noted. First, as this study was observational, we could not exclude the possibility of residual confounding and reverse causation, although we adjusted for multiple potential confounders. Second, data on lifestyle changes over time were not available, so the trajectory of PA or sleep traits on the incident PD could not be examined. Third, the current analyses were predominantly performed in European populations, and so further studies are needed to confirm our findings among other populations or ethnic groups.

## Conclusions

In conclusion, we found that both high PA and ideal sleep pattern were independently associated with lower risk of incident PD risk, with no statistically significant evidence of interaction. However, those with both high PA and ideal sleep pattern had the lowest PD risk, suggesting that improving PA levels and sleep quality may be promising intervention targets and may open new opportunities for the prevention and management of PD.

### Electronic supplementary material

Below is the link to the electronic supplementary material.

Supplementary Material 1

Supplementary Material 2

## Data Availability

Researchers registered with UK Biobank can apply for access to the database by completing an application. (https://www.ukbiobank.ac.uk/enable-your-research/apply-for-access). The code is available from the corresponding author upon reasonable request.

## Abbreviations

BMI: Body mass index
CI: Confidence interval
DBP: Diastolic blood pressure
HR: Hazard ratio
IPAQ: International Physical Activity
MET: Metabolic equivalent
PA: Physical activity
PD: Parkinson’s disease
SBP: Systolic blood pressure
