# Supplementary material for: Physical activity and sleep pattern in relation to incident Parkinson’s disease: a cohort study
Source: Int J Behav Nutr Phys Act. 2024 Feb 14;21:17. doi: 10.1186/s12966-024-01568-9 (PMC10867998; doi:10.1186/s12966-024-01568-9)
Supplement: Supplementary file 2 — Supplementary Material 2 [file 12966_2024_1568_MOESM2_ESM.pdf]

Supplementary Table S1. Code lists used in the UK Biobank study to identify Parkinson's disease cases.

**ICD-9**
Paralysis agitans: 332.0
Secondary parkinsonism: 332.1
Other degenerative diseases of basal ganglia: 333.0
**ICD-10**
Parkinson's disease: G20
Secondary parkinsonism: G21
Malignant neuroleptic syndrome: G21.0
Other drug induced secondary parkinsonism: G21.1
Secondary parkinsonism due to other external agents: G21.2
Post encephalitic parkinsonism: G21.3
Vascular parkinsonism: G21.4
Other secondary parkinsonism: G21.8
Secondary parkinsonism unspecified: G21.9
Parkinsonism in diseases specified elsewhere: G22
Hallervorden-Spatz disease: G23.0
Progressive Supranuclear Palsy: G23.1
Multiple system atrophy, parkinsonian type [MSA-P]: G23.2
Multiple system atrophy, cerebellar type [MSA-C]: G23.3
Other specified degenerative diseases of basal ganglia (Calcification of basal ganglia Neurogenic orthostatic hypotension [ShyDrager]: G23.8
Degenerative diseases of basal ganglia, unspecified: G23.9
Extrapyramidal and movement disorder, unspecified: G25.9
Extrapyramidal and movement disorders in diseases classified elsewhere: G26
Multi-system degeneration: G90.3
**Self-Report**
Parkinson's disease

Supplementary Table S2. Details on classifying three levels of PA by the UK Biobank.

Levels of PA	Classification criteria
Low: Categories 1<br>Either of the 2 criteria	● No activity is reported<br>● Some activity is reported but not enough to meet Categories 2 or 3
Medium: Categories 2	● 5 or more days of any combination of walking, moderate-intensity or vigorous intensity activities achieving a minimum of at least 600 MET-minutes/week.
High: Categories 3	● 7 or more days of any combination of walking, moderate- or vigorous-intensity activities accumulating at least 3000 MET-minutes/week.

Based on the scoring system of UK Biobank, baseline PA levels were grouped into low (0 to <600 MET-mins/week), medium (600 to <3000 MET-mins/week) and high (≥3000 MET-mins/week) according to instruction of the UK Biobank. PA, physical activity

Supplementary Table S3. The scoring system of sleep.

**Characteristics**	**UK BioBank Code**	**UK BioBank Questionnaire**	**Healthy Answer (%)**	**Unhealthy Answer (%)**
Chronotype	1180	Do you consider yourself to be?	Definitely a "morning" person; More a "morning" than "evening" person. (57%)	More an "evening" than a "morning person; Definitely an "evening" person. (43%)
Sleep Duration	1160	About how many hours sleep do you get in every 24 hours? (please include naps)	7-8 hr/d. (69%)	<7 or >=9 hr/d. (31%)
Insomnia	1200	Do you have trouble falling asleep at night or do you wake up in the middle of the night?	Never/rarely; Sometimes (73%)	Usually (27%)
Snoring	1210	Does your partner or a close relative or friend complain about your snoring?	No (60%)	Yes (40%)
Daytime Sleepiness	1220	How likely are you to doze off or fall asleep during the daytime when you don't mean to? (e.g. when working, reading or driving)	Never/rarely; Sometimes (98%)	Often; All the Time (2%)

For further information, please refer to the UK Biobank data showcase https://biobank.ndph.ox.ac.uk/showcase/search.cgi

Supplementary Table S4. Baseline participants' characteristics by PD status.

	**Without incident PD (99.4%)**	**With incident PD (0.6%)**	***P* value**
Age, y	56.2±8.1	62.9±5.4	<0.001
Men	158269 (46.9%)	1287 (64.5%)	<0.001
White	320229 (94.8%)	1919 (96.1%)	0.072
Townsend deprivation index	-1.5±3.0	-1.4±3.0	0.477
BMI, kg/m^2	27.3±4.7	27.7±4.5	<0.001
Current smoker	34288 (10.2%)	130 (6.5%)	<0.001
PA levels, MET-mins/week	2660.7±2706.6	2446.4±2526.2	<0.001
PA categories*			0.004
Low	62307 (18.5%)	402 (20.1%)	
Medium	170965 (50.6%)	1042 (52.2%)	
High	104398 (30.9%)	552 (27.7%)	
Healthy sleep score	3.6±1.0	3.5±1.0	<0.001
Sleep pattern†			<0.001
Poor	44768 (13.3%)	336 (16.8%)	
Ideal	292902 (86.7%)	1660 (83.2%)	
Sedentary time, h/d	4.8±2.4	5.0±2.3	0.009
SBP, mmHg	139.4±19.6	143.2±19.9	<0.001
DBP, mmHg	82.2±10.7	81.9±10.3	<0.001
Healthy diet score	2.9±1.4	3.1±1.4	<0.001
Alcohol consumption, g/d	15.2±19.0	14.9±19.5	0.525
Never drink coffee	73756 (21.8%)	427 (21.4%)	0.917
Never drink tea	49312 (14.6%)	254 (12.7%)	0.101
Hypertensive medication use	67425 (20.0%)	688 (34.5%)	<0.001
Diabetes	19474 (5.8%)	228 (11.4%)	<0.001

Continuous variables are described as means ± standard deviation, and categorical variables are described as numbers and percentages.

BMI, body mass index; DBP, diastolic blood pressure; PA, physical activity; SBP, systolic blood pressure; PD, Parkinson's disease

* PA was categorized as low (<600 MET-mins/week), medium (600 to <3000 MET-mins/week) and high (≥3000 MET-mins/week)

† Participants were scored from 0 to 5, according to their number of the healthy sleep characteristics and were categorized into two groups: "ideal sleep pattern" (≥3 sleep scores) and "poor sleep pattern" (0-2 sleep scores)

Supplementary Table S5. Hazard ratios for the associations of specific sleep characteristics with risk of Parkinson's disease.

	**Prevalence**	**Multivariable-adjusted [HR (95% CI)]**	***P* value**
Early chronotype	63.0%	0.91 (0.83, 0.99)	0.036
Adequate sleep duration	68.8%	0.80 (0.73, 0.88)	<0.001
Not usually insomnia	72.9%	1.01 (0.91, 1.11)	0.895
No snoring	62.8%	0.90 (0.82, 0.99)	0.028
No frequent daytime sleepiness	97.4%	0.68 (0.56, 0.84)	<0.001

Multivariable model was adjusted for age, sex, ethnicity, Townsend deprivation index, smoking status, BMI, SBP, DBP, antihypertensive medication use, sedentary time, healthy diet score, alcohol consumption, coffee consumption, tea consumption, and diabetes.

CI, confidence interval; HR, hazard ratio

Supplementary Table S6. Hazard ratios for the joint associations of PA and healthy sleep category with risk of Parkinson's disease, stratified by age and sex.

***P*-interaction = 0.231**	**Age <65 y**		**Age ≥65 y**	
	**Events/person-y**	**Multivariable-adjusted HR (95% CI)**	**Events/person-y**	**Multivariable-adjusted HR (95% CI)**
**Poor sleep pattern**				
Low PA	60/110,049	Reference	33/18,755	Reference
Medium PA	89/204,017	0.81 (0.58, 1.12)	71/42,549	0.93 (0.62, 1.41)
High PA	44/116,283	0.71 (0.48, 1.06)	39/26,305	0.82 (0.51, 1.30)
**Ideal sleep pattern**				
Low PA	170/504,897	0.68 (0.50, 0.91)	139/87,990	0.89 (0.61, 1.30)
Medium PA	447/1,447,742	0.62 (0.47, 0.82)	435/300,636	0.81 (0.56, 1.15)
High PA	236/859,507	0.53 (0.39, 0.70)	233/212,349	0.60 (0.42, 0.87)
***P*-interaction = 0.358**	**Women**		**Men**	
	**Events/person-y**	**Multivariable-adjusted HR (95% CI)**	**Events/person-y**	**Multivariable-adjusted HR (95% CI)**
**Poor sleep pattern**				
Low PA	30/64,473	Reference	63/64,331	Reference
Medium PA	52/127,336	0.83 (0.53, 1.30)	108/119,231	0.85 (0.62, 1.16)
High PA	30/67,931	0.87 (0.52, 1.45)	53/74,657	0.69 (0.48, 0.99)
**Ideal sleep pattern**				
Low PA	102/319,637	0.73 (0.48, 1.10)	207/273,249	0.76 (0.57, 1.01)
Medium PA	320/966,547	0.73 (0.50, 1.06)	562/781,831	0.67 (0.51, 0.87)
High PA	175/554,113	0.63 (0.43, 0.94)	294/517,743	0.51 (0.39, 0.67)

Multivariable model was adjusted for age, sex, ethnicity, Townsend deprivation index, smoking status, BMI, SBP, DBP, antihypertensive medication use, sedentary time, healthy diet score, alcohol consumption, coffee consumption, tea consumption, and diabetes.

CI, confidence interval; HR, hazard ratio; PA, physical activity

Supplementary Table S7. Sensitivity analysis for the joint associations of PA and healthy sleep category with risk of Parkinson's disease.

**Exclusion of participants with missing data**	**Events/person-y**	**Multivariable-adjusted HR (95% CI)**
**Poor sleep pattern**		
Low PA	89/125,187	Reference
Medium PA	147/241,611	0.80 (0.62, 1.05)
High PA	81/139,372	0.75 (0.56, 1.02)
**Ideal sleep pattern**		
Low PA	300/580,665	0.75 (0.59, 0.96)
Medium PA	858/1,718,579	0.69 (0.55, 0.86)
High PA	460/1,053,193	0.55 (0.44, 0.70)
**Redefining sleep patterns by excluding insomnia***	**Events/person-y**	**Multivariable-adjusted HR (95% CI)**
**Poor sleep pattern**		
Low PA	180/263,542	Reference
Medium PA	361/564,758	0.87 (0.73, 1.04)
High PA	174/327,108	0.70 (0.57, 0.87)
**Ideal sleep pattern**		
Low PA	222/458,149	0.72 (0.59, 0.88)
Medium PA	681/1,430,188	0.68 (0.57, 0.80)
High PA	378/887,338	0.55 (0.46, 0.66)
**Exclusion of participants who developed Parkinson disease within the first 2 year of follow-up**	**Events/person-y**	**Multivariable-adjusted HR (95% CI)**
**Poor sleep pattern**		
Low PA	77/128,447	Reference
Medium PA	137/246,192	0.87 (0.66, 1.15)
High PA	74/142,397	0.80 (0.58, 1.10)
**Ideal sleep pattern**		
Low PA	275/591,880	0.80 (0.62, 1.03)
Medium PA	816/1,746,483	0.77 (0.60, 0.97)
High PA	435/1,070,600	0.61 (0.48, 0.78)

Multivariable model was adjusted for age, sex, ethnicity, Townsend deprivation index, smoking status, BMI, SBP, DBP, antihypertensive medication use, sedentary time, healthy diet score, alcohol consumption, coffee consumption, tea consumption, and diabetes. CI, confidence interval; HR, hazard ratio; PA, physical activity.

*Poor sleep pattern was defined as a sleep core of ≤2, and ideal sleep pattern was defined as a sleep score of >2.

Supplementary Figure S1. Flowchart of the study population selection.

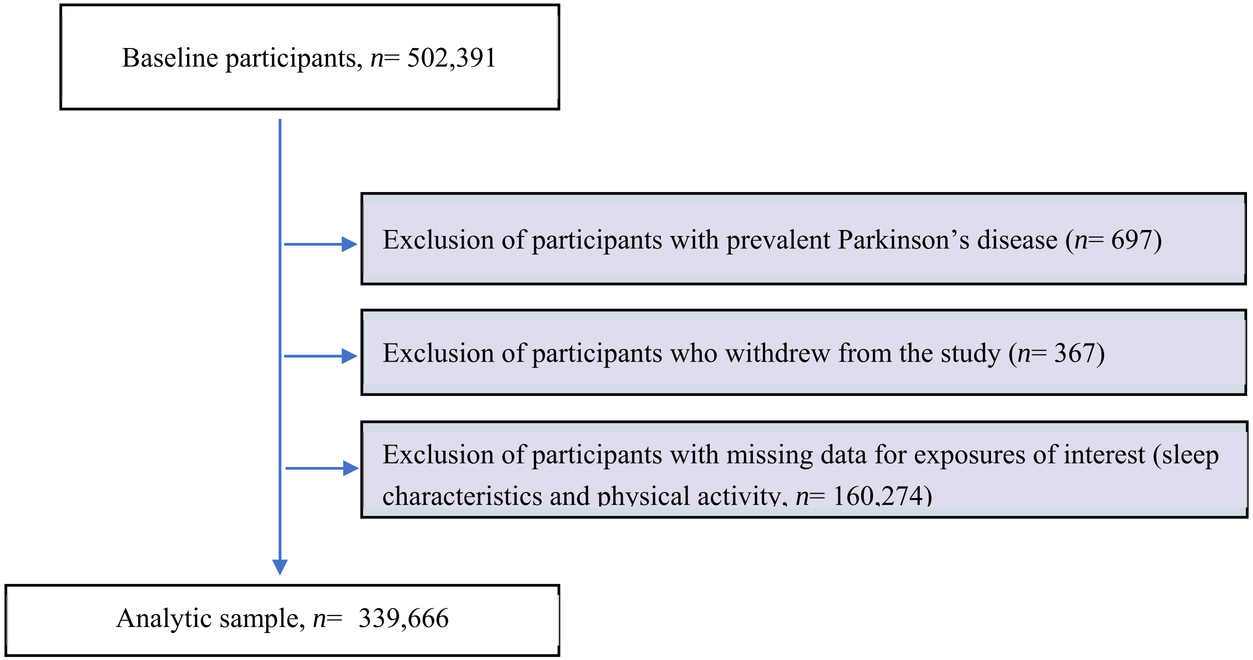

Supplementary Figure S2. Kaplan-Meier plot showing risk of Parkinson's disease by PA and sleep pattern combined.

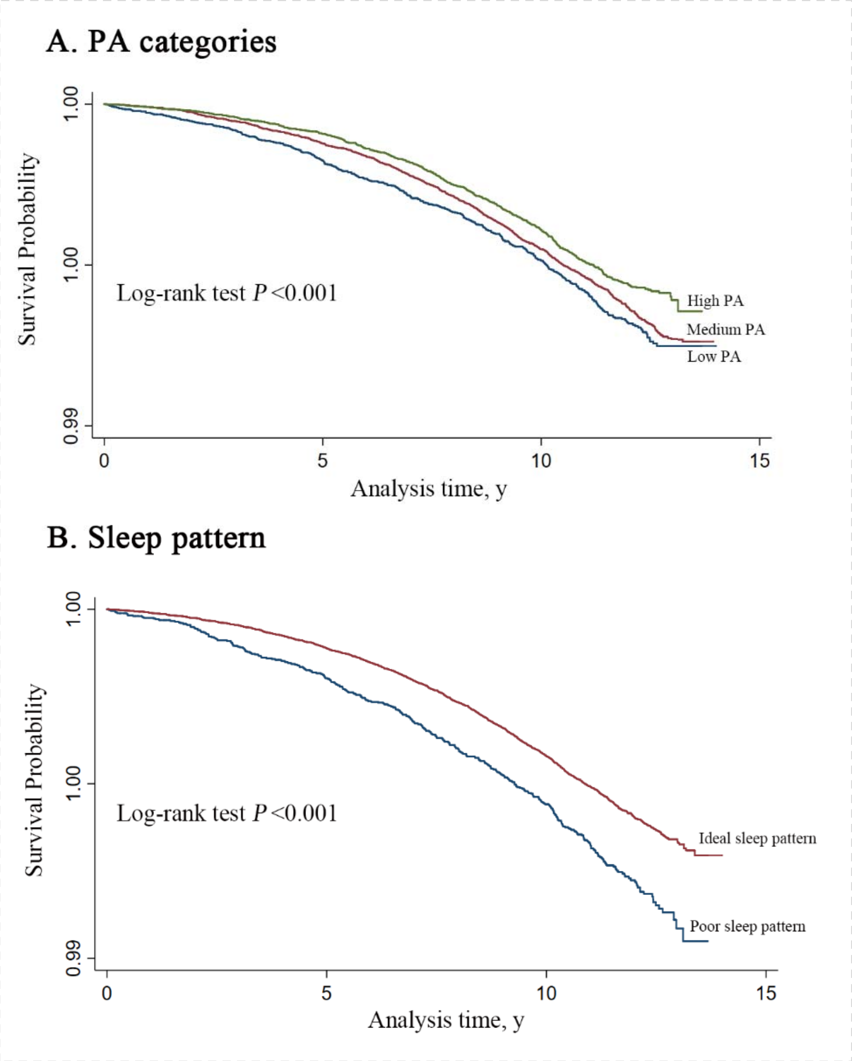

PA, physical activity

Supplementary Figure S3. Association between PA levels and healthy sleep score as a continuous scale and incident Parkinson's disease.

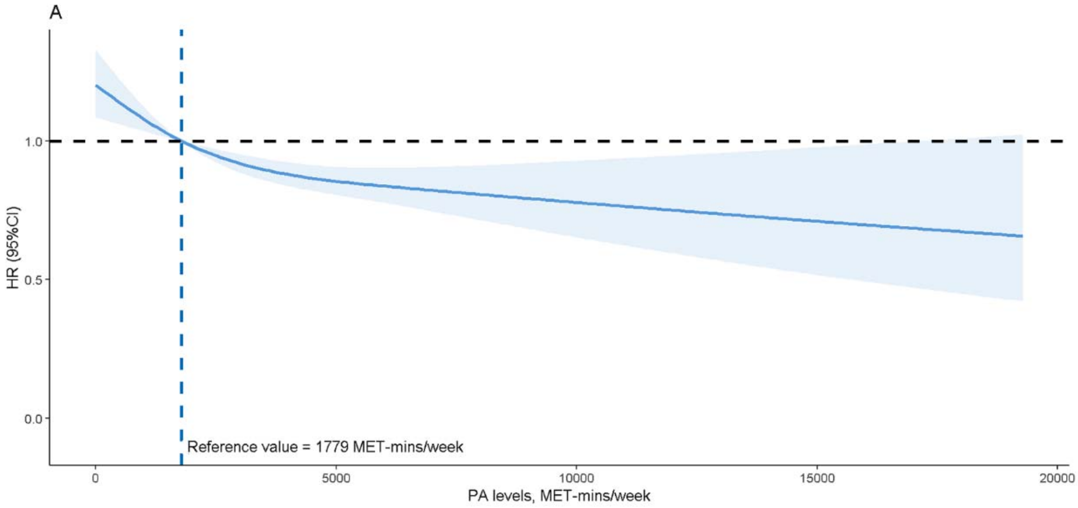

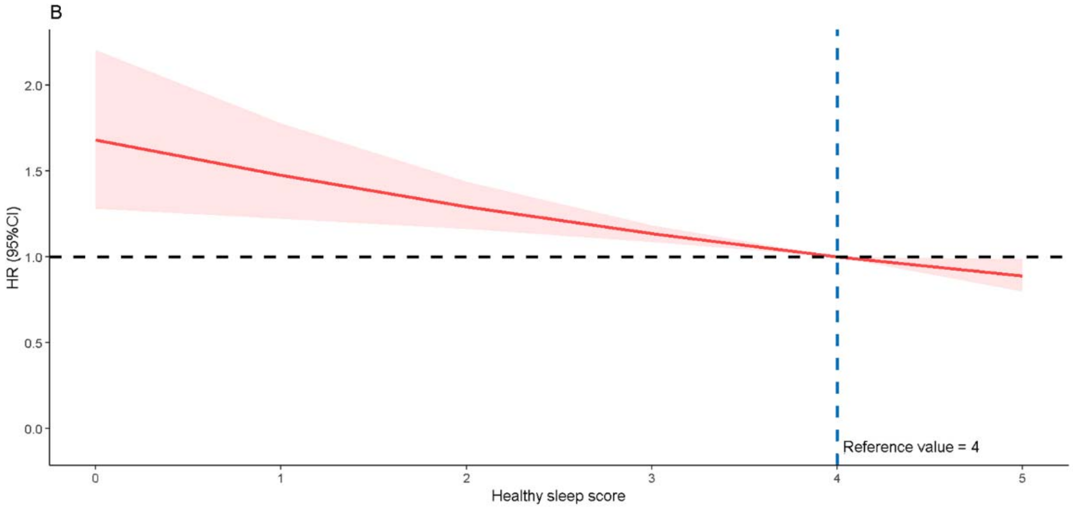

The hazard ratio (solid line) and 95% confidence interval (shaded region) were obtained from the Cox regression using restricted cubic splines. Multifactorial adjustments were made for age, sex, ethnicity, Townsend deprivation index, smoking status, BMI, SBP, DBP, antihypertensive medication use, sedentary time, healthy diet score, alcohol consumption, coffee consumption, tea consumption, and diabetes. PA and healthy sleep score were mutually adjusted for each other. Restricted cubic splines with three knots were used to flexibly model the non-linear relationship. For PA, we set 1779 MET-min/week as the reference; and for sleep scores, we set the score of 4 as the reference.

Supplemental Figure S4. Kaplan-Meier plot showing risk of Parkinson's disease by individual sleep characteristics.

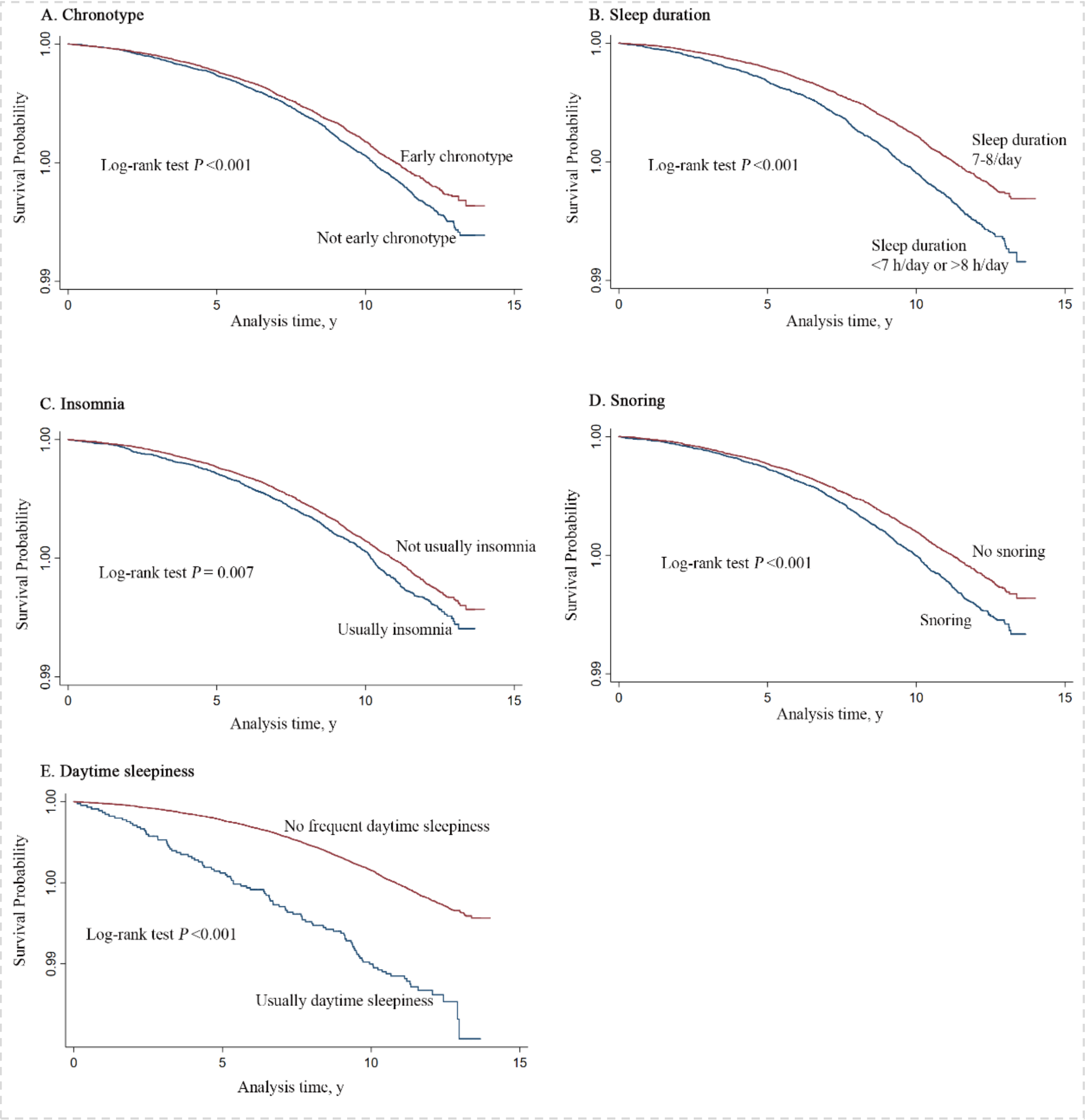

Supplemental Figure S5. Kaplan-Meier plot showing risk of Parkinson's disease by PA and sleep pattern combined.

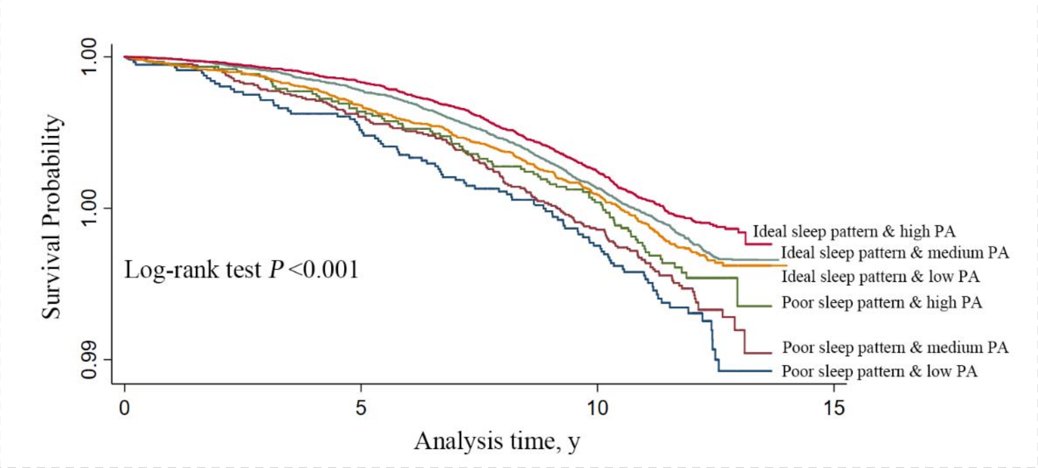

PA, physical activity
